# Supplementary material for: The microtubule GTP-tubulin cap size is modulated during cell division
Source: Mol Biol Cell. 2026 Apr 6;37(5):br14. doi: 10.1091/mbc.E26-01-0054 (PMC13322201; doi:10.1091/mbc.E26-01-0054)
Supplement: Supplementary file 2 [file mbc-37-br14-s001.pdf]

Supplemental Materials

Molecular Biology of the Cell

Cassidy *et al*.

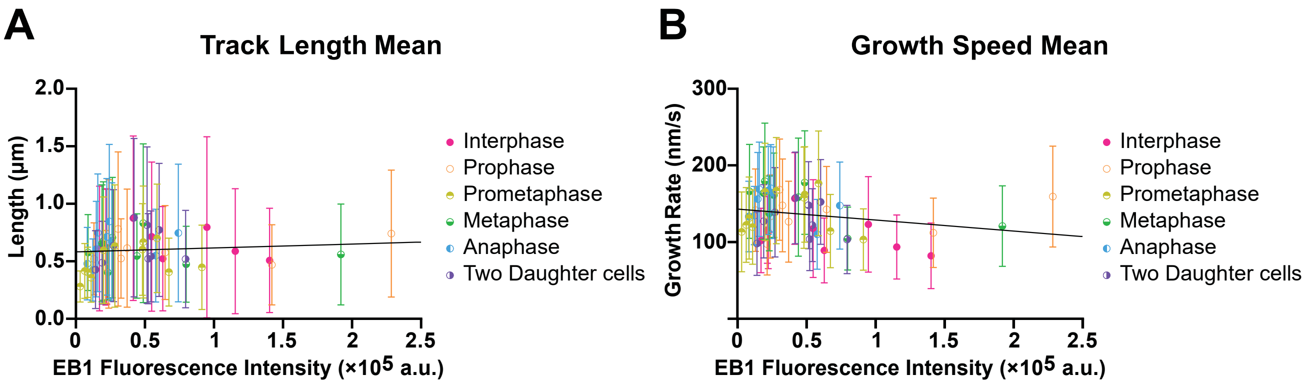
A
Track Length Mean
Length (µm)
2.0
1.5
1.0
0.5
0.0
0
0.5
1
1.5
2
2.5
EB1 Fluorescence Intensity (×10^5 a.u.)
Interphase
Prophase
Prometaphase
Metaphase
Anaphase
Two Daughter cells
B
Growth Speed Mean
Growth Rate (nm/s)
300
200
100
0
0.5
1
1.5
2
2.5
EB1 Fluorescence Intensity (×10^5 a.u.)
Interphase
Prophase
Prometaphase
Metaphase
Anaphase
Two Daughter cells

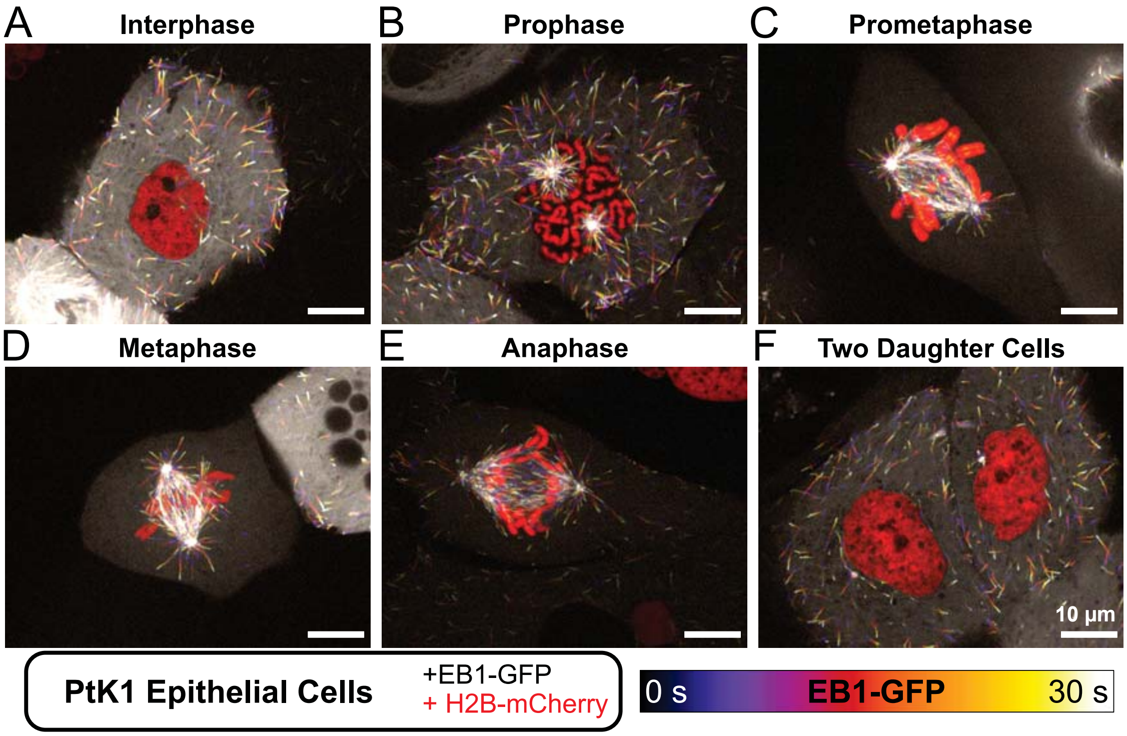
A
Interphase
B
Prophase
C
Prometaphase
D
Metaphase
E
Anaphase
F
Two Daughter Cells
10 µm
PtK1 Epithelial Cells
+EB1-GFP
+ H2B-mCherry
0 s
EB1-GFP
30 s

Figure S1. Microtubule growth parameters are not affected by EB1-GFP expression levels in LLC-PK1 cells. (A) Mean microtubule growth length plotted against corrected total cell fluorescence (CTCF) of EB1-GFP. (B) Mean microtubule growth speed plotted against CTCF of EB1-GFP. Microtubule dynamic parameters were quantified using plusTipTracker (Applegate et al., 2011). Each data point represents a single cell; error bars indicate standard deviation (SD). N = 56 cells. Linear regression analysis revealed no significant correlation between EB1-GFP expression level and either growth length (A, $p = 0.26$) or growth speed (B, $p = 0.99$).

Figure S2. EB1 comets in PtK1 cells throughout the cell cycle. (A-F) Representative images of temporally-colored maximum intensity projection of EB1-GFP comets over time in PtK1 cells stably expressing EB1-GFP and transiently expressing mCherry-H2B during a 30-second movie. Chromosomes are visualized at the starting timepoint via acquisition of a single frame of mCherry-H2B signal shown in orange. Cells were classified into different cell cycle stages based on chromosome organization: (A) interphase, (B) prophase, (C) prometaphase, (D) metaphase, (E) anaphase, and (F) two daughter cells. EB1-GFP intensity across all representative images is scaled identically.
