# Supplementary material for: The microtubule GTP-tubulin cap size is modulated during cell division
Source: Mol Biol Cell. 2026 Apr 6;37(5):br14. doi: 10.1091/mbc.E26-01-0054 (PMC13322201; doi:10.1091/mbc.E26-01-0054)
Supplement: Supplementary file 3 [file reviewer_reportE26-01-0054.pdf]

The microtubule GTP-tubulin cap size is modulated during cell division

Anna Cassidy, Dylan Burnette, and Marija Zanic

Corresponding author(s): Marija Zanic, Vanderbilt University

Review Timeline:

Submission Date:	2026-01-29
Editorial Decision:	2026-02-13
Revision Received:	2026-02-27
Accepted:	2026-03-02

Editor-in-Chief: John Aitchison

Transaction Report:

(Note: With the exception of the correction of typographical or spelling errors that could be a source of ambiguity, letters and reports are not edited. The original formatting of letters and referee reports may not be reflected in this compilation.)

RE: Manuscript #E26-01-0054
TITLE: "The microtubule GTP-tubulin cap size is modulated during cell division"

Dear Dr. Zanic:

I enjoyed reading your manuscript, and the reviewers are also positive. Please address constructively all their comments, and I will see if I can make a decision myself or send it to one of the reviewers for the second look.

Thank you again for your contribution to MBoC.

Sincerely,
Alexander Mogilner
Monitoring Editor
Molecular Biology of the Cell

Dear Dr. Zanic,

The review of your manuscript, referenced above, is now complete. The Monitoring Editor has decided that your manuscript requires minor revisions before it can be published in Molecular Biology of the Cell, as described in the Monitoring Editor's decision letter above and the reviewer comments (if any) below.

A reminder: Please do not contact the Monitoring Editor directly regarding your manuscript. If you have any questions regarding the review process or the decision, please contact the MBoC Editorial Office (mboc@ascb.org).

When submitting your revision include an authors' response letter that details, point-by-point, how the Monitoring Editor's and reviewers' comments have been addressed. (The file type for this letter must be "response letter"; do not include your response to the Monitoring Editor and reviewers in a "cover letter.") Please bear in mind that your response letter will be published with your paper if it is accepted, unless you have opted out of publishing the review history.

Authors are allowed 15 days to submit a revision. If this time period is inadequate, please contact us immediately at mboc@ascb.org.

In preparing your revised manuscript, please follow the instructions in the Information for Authors (www.molbiolcell.org/info-for-authors). In particular, to prepare for the possible acceptance of your revised manuscript, submit final, publication-quality figures with your revision as described.

To submit the response letter, revised version, and figures, please use this link (please enable cookies, or cut and paste URL):
Link Not Available

MBoC offers the option to publish your work with immediate open access. Open access can increase the discoverability and usability of your research. If you would like to publish your paper with immediate open access but did not select this option during initial submission, please contact us at mbc@ascb.org.

Authors of Articles and Brief Communications whose manuscripts have returned for minor revision ("provisional acceptance") are encouraged to create a short video abstract to accompany their article when it is published. These video abstracts, known as Science Sketches, are up to 2 minutes long and will be published on YouTube and then embedded in the article abstract. Science Sketch Editors on the MBoC Editorial Board will guide you as you prepare your video. Information about how to prepare and submit a video abstract is available at www.molbiolcell.org/science-sketches. Please contact mboc@ascb.org if you are interested in creating a Science Sketch.

Thank you for submitting your manuscript to MBoC. Please do not hesitate to contact this office if you have any questions.

Sign up for MBoC's new content alerts here https://tinyurl.com/3bcsyk3e

Sincerely,

Eric Baker
Journal Production Manager
MBoC Editorial Office

mbc@ascb.org

Reviewer #1 (Remarks to the Author):

The manuscript by Cassidy et al addresses an open question in the cytoskeleton field - how do polymer dynamics change as cells progress through the cell cycle. For microtubules, prior research has shown that microtubule dynamics can change under different contexts. Here, the authors examine microtubule dynamics at different cell cycle stages using EB1-GFP as a proxy for the rate of GTP hydrolysis. Using careful analysis of live imaging, the authors measure the microtubule growth rate and the EB1-GFP cap size. They show that GTP-cap size varies across the cell cycle and that the changes are not spatially isolated. And they show this in two diffent cell lines. These findings suggest that the inherent GTP hydrolysis rate of tubulin in the lattice is regulated in global manner. The manuscript is well-written, the data are carefully considered and analyzed, and the findings are important for the field. I have just a few questions that may improve clarity.

1) Is it clear that a decrease in GTP-cap size in prometaphase and anaphase means a faster rate of GTP hydrolysis in the lattice? Or could it be a faster rate of Pi release after GTP hydrolysis? Since EB1 prefers a compacted lattice, this seems inconsistent with representing a pre-hydrolysis GTP cap.

2) Since these are stable cell lines, why does the level of EB1-GFP vary across cells?

3) For the representative images, it would be helpful to know whether these were selected based on having similar CTCF. If not, could the CTCF of the representative cells be included in the figure or legend?

4) How was microtubule growth length calculated in Fig S1? Is this total length of microtubule growth during the imaging period? If so, its important to note that the total imaging time was kept constant. Or is it average microtubule growth length from varied imaging times?

5) For Fig 1G (and others), have the authors checked whether the different colors are visible to those who are color blind?

Reviewer #2 (Remarks to the Author):

The "GTP cap" is a major determinant of microtubule stability and is formed by tubulin subunits that have not yet hydrolyzed their β-tubulin-associated GTP. It is well known that microtubule dynamics change throughout the cell cycle and that MAPs (including XMAP215 and kinesin-13/MCAK) are largely responsible for these changes. However, a role for regulation of GTP cap size has not been explored. In this manuscript, Cassidy et al. present an analysis of GTP cap size throughout the cell cycle by measuring the comet lengths of EB1, a well-established marker of the GTP cap. The authors report the interesting finding that the GTP cap decreases in size during cell division. This decrease cannot be accounted for by differences in microtubule growth rates or by increased levels of EB1 (the authors use an LLC-PK1 cell line that stably expresses exogenous EB1-GFP). Moreover, the authors observe a similar change in PtK1 cells that stably express EB1-GFP, suggesting that a reduction in GTP cap size occurs broadly among animal cells. Altogether, this work suggests that dividing cells decrease the size of the GTP cap, which may be an important mechanism underlying the ~10-fold increase in microtubule catastrophes observed during mitosis. This work provides strong justification for future studies aimed at identifying proteins that modulate GTP cap size, for example, proteins that stimulate or inhibit the GTPase activity of β-tubulin. I recommend publication of this work in Molecular Biology of the Cell, with only minor adjustments.

Major comments

1. It would be useful to more fully describe how the authors selected spindle-associated EB1 comets for analysis. Were EB1 comets associated with kinetochores, k-fibers, or interpolar microtubules? Since cells only contained one additional marker (H2B), I know that it would be difficult to unambiguously assign EB1 comets to specific spindle microtubule populations. However, details about how EB1 comets were chosen would be helpful, because microtubule plus end sub-populations are very likely to have different plus end behaviors.

2. It would be useful if the authors described how they were able to eliminate focal plane drift (i.e., movement of a plus end into an adjacent focal plane) as a possible explanation for comet length shortening.

3. The Conclusions section of the manuscript is strong but could be improved, e.g., by adding text recognizing that microtubule growth rates contribute to spindle length regulation (e.g., Lacroix et al. (2018); Reber et al. (2013)) as well as chromosome segregation fidelity (Ertych et al. (2014)). GTP cap size may be an important aspect of these biological phenomena. Moreover, it has been suggested that Cyclin A and Cyclin B control spindle microtubule dynamics during prometaphase versus metaphase (Kabeche and Compton (2013)). It would be interesting if the differences in GTP cap size was directly regulated by the core cell

cycle machinery.

4. It would be very helpful to insert a figure similar to Figure 1 for the analysis done in PtK1 cells.

Minor comments
1. A scale bar is missing from Figure 1F.

Cassidy et al. The microtubule GTP-tubulin cap size is modulated during cell division

Response to Reviewers

We thank the reviewers for their constructive feedback. Below, we elaborate on all of the revisions made and provide a point-by-point response to reviewers' comments. Overall, we feel that our manuscript has improved in presentation and clarity based on the reviewers' feedback.

Reviewer #1 (Remarks to the Author):

The manuscript by Cassidy et al addresses an open question in the cytoskeleton field - how do polymer dynamics change as cells progress through the cell cycle. For microtubules, prior research has shown that microtubule dynamics can change under different contexts. Here, the authors examine microtubule dynamics at different cell cycle stages using EB1-GFP as a proxy for the rate of GTP hydrolysis. Using careful analysis of live imaging, the authors measure the microtubule growth rate and the EB1-GFP cap size. They show that GTP-cap size varies across the cell cycle and that the changes are not spatially isolated. And they show this in two different cell lines. These findings suggest that the inherent GTP hydrolysis rate of tubulin in the lattice is regulated in global manner. The manuscript is well-written, the data are carefully considered and analyzed, and the findings are important for the field. I have just a few questions that may improve clarity.

We thank the reviewer for their enthusiastic endorsement of our work.

1. Is it clear that a decrease in GTP-cap size in prometaphase and anaphase means a faster rate of GTP hydrolysis in the lattice? Or could it be a faster rate of Pi release after GTP hydrolysis? Since EB1 prefers a compacted lattice, this seems inconsistent with representing a pre-hydrolysis GTP cap.

The reviewer raises a valid point. The term 'GTP-cap' is synonymous with the nucleotide-dependent stabilizing structure at growing microtubule ends. Extensive in vitro reconstitution and structural studies (particularly from the Surrey and Nogales labs) have established that EB proteins recognize and mark this nucleotide-dependent stabilizing structure. However, the exact nucleotide composition of the GTP-cap, particularly with respect to the relative amounts of GTP vs GDP-Pi tubulin (in which hydrolysis has occurred, but the phosphate has not yet been released), is not fully understood. Therefore, the observed decrease in GTP-cap size could be a consequence of a faster Pi release step following hydrolysis, rather than strictly the initial GTP hydrolysis step. To acknowledge this point, we have amended the text in our Discussion as follows:

Lines 348-350: "Thus, the cell-cycle dependent modulation of the GTP-cap size for a given growth rate observed in our experiments could be explained by mitosis-specific regulation of the GTP-hydrolysis **or phosphate-release** rate."

2. Since these are stable cell lines, why does the level of EB1-GFP vary across cells?

This stable cell line was originally generated by Piehl and Cassimeris (2003). The cell line was developed by selecting and screening colonies, rather than individual cell clones; therefore, there is some inherent heterogeneity in the EB1-GFP expression levels between cells. However, based on our experience, the overall variability in expression levels of EB1-GFP in the stably expressing cells appears moderate compared with typical transient overexpression conditions. Nevertheless, to reduce the potential for any adverse overexpression effects, the cells were first prescreened to identify those displaying moderate expressions levels suitable for our analysis. Following this prescreening, we performed a rigorous quantitative evaluation of potential overexpression effects on microtubule dynamics for all cells included in the analysis, as shown in Figure S1.

3. For the representative images, it would be helpful to know whether these were selected based on having similar CTCF. If not, could the CTCF of the representative cells be included in the figure or legend?

We appreciate the reviewer's suggestion to clarify how the representative images were selected. Indeed, the cells shown in Figure 1 have similar CTCF values, ranging between 0.2-0.5 × 10^5 a.u., selected as representative examples consistent with the median (0.28 × 10^5 a.u.) and the mean (0.45 ± 0.05 × 10^5 a.u., SEM) of the overall CTCF value distribution. Importantly, the EB1 intensity across all representative images is scaled identically to enable direct comparison. To clarify this point, we have added the following text to the Figure 1 caption:

Line 143-144: "EB1-GFP intensity across all representative images is scaled identically."

4. How was microtubule growth length calculated in Fig S1? Is this total length of microtubule growth during the imaging period? If so, its important to note that the total imaging time was kept constant. Or is it average microtubule growth length from varied imaging times?

We thank the reviewer for highlighting this point of potential confusion. Although individual EB tracks vary in duration, the total imaging time was kept constant. Briefly, EB tracks are extracted from 30 s videos using the established plusTipTracker approach (Applegate et al., 2011). Tracks present in the first or last frame are excluded (by selecting *"remove tracks at the beginning and end of the movie"*) to ensure that only complete growth events are analyzed. The plusTipTracker quantifies microtubule growth lengths as the cumulative EB comet displacement over the duration of each track, calculated from frame-to-frame displacements. Thus, this metric reflects the total microtubule growth for each track during a single growth event. Growth lengths are then averaged across all tracks within a given cell imaged during the 30 s acquisition period. Each data point in Figure S1 represents the average track length obtained using this procedure, along with its standard deviation.

To clarify this point and provide more details on the parameters used in this analysis, we have now expanded the Methods section "Determination of the effect of EB1 overexpression on microtubule dynamics" as follows:

Line 435-446: "All EB comets were extracted from 30 s time-lapse videos. Comets were detected using anisotropic Gaussian filtering (α = 0.01) with a minimum separation distance of 5 pixels to prevent duplicate detections. Tracking was performed using the Microtubule Plus-End Dynamics application, with segment merging and splitting disabled to minimize tracking errors. To apply the most stringent tracking criteria, gap closure was set to 0 and the minimum track length was set to 3 frames. For frame-to-frame linking, lower and upper displacement bounds of 2 and 4 pixels, respectively, were used. The Brownian search radius multiplication factor was set to 3, and nearest-neighbor linking was applied. Maximum forward and reverse tracking angles were set to 30° and 10°, respectively, with a maximum shrinkage factor of 1.5 and a fluctuation radius of 1 pixel. Tracks present in the first or last frame were excluded (using the "remove tracks at the beginning and end of the movie" option) to ensure that only complete growth events were analyzed. The resulting tracks were subsequently analyzed using the Microtubule Dynamics Classification module."

5. For Fig 1G (and others), have the authors checked whether the different colors are visible to those who are color blind?

We thank the reviewer for raising this important point. We have updated all graphs containing multiple colors (panels 2G, 4G and Figure S1) to also include distinct symbols for each phase, making the figures accessible and interpretable for readers with color blindness.

Reviewer #2 (Remarks to the Author):

The "GTP cap" is a major determinant of microtubule stability and is formed by tubulin subunits that have not yet hydrolyzed their β-tubulin-associated GTP. It is well known that microtubule dynamics change throughout the cell cycle and that MAPs (including XMAP215 and kinesin-13/MCAK) are largely responsible for these changes. However, a role for regulation of GTP cap size has not been explored. In this manuscript, Cassidy et al. present an analysis of GTP cap size throughout the cell cycle by measuring the comet lengths of EB1, a well-established marker of the GTP cap. The authors report the interesting finding that the GTP cap decreases in size during cell division. This decrease cannot be accounted for by differences in microtubule growth rates or by increased levels of EB1 (the authors use an LLC-PK1 cell line that stably expresses exogenous EB1-GFP). Moreover, the authors observe a similar change in PtK1 cells that stably express EB1-GFP, suggesting that a reduction in GTP cap size occurs broadly among animal cells. Altogether, this work suggests that dividing cells decrease the size of the GTP cap, which may be an important mechanism underlying the ~10-fold increase in microtubule catastrophes observed during mitosis. This work provides strong justification for future studies aimed at identifying

proteins that modulate GTP cap size, for example, proteins that stimulate or inhibit the GTPase activity of β-tubulin. I recommend publication of this work in Molecular Biology of the Cell, with only minor adjustments.

We thank the reviewer for their enthusiastic support of our work.

Major comments

1. It would be useful to more fully describe how the authors selected spindle-associated EB1 comets for analysis. Were EB1 comets associated with kinetochores, k-fibers, or interpolar microtubules? Since cells only contained one additional marker (H2B), I know that it would be difficult to unambiguously assign EB1 comets to specific spindle microtubule populations. However, details about how EB1 comets were chosen would be helpful, because microtubule plus end sub-populations are very likely to have different plus end behaviors.

We appreciate the reviewer's request to clarify our methods. To identify spindle-associated EB1 comets for analysis, we initially selected all discernible individual EB1 tracks within the spindle. The high spatiotemporal resolution of our imaging allowed us to resolve individual comets (Figure 3A shows an example spindle with lines indicating identified tracks). Tracks were required to have a minimum duration of 4 seconds. All data were subsequently subjected to rigorous quality control based on our ability to quantitatively measure both growth rate and comet length with high confidence, which led to the exclusion of many tracks. This approach allows for a relatively lenient initial track selection while ensuring that only high-confidence measurements are included in the final analysis. As noted by the reviewer, our live-cell imaging did not include additional markers that would allow us to unambiguously assign EB1 comets to specific spindle microtubule subpopulations (e.g., kinetochores, k-fibers, or interpolar microtubules), though we agree this would be valuable for future work.
To provide further clarification on how EB1 comets were chosen for analysis, we have added the following text to or Methods section:

Lines 471-473: All discernible individual EB1-GFP tracks in each cell were identified and considered for tracking analysis. Classification of astral vs. spindle microtubules in metaphase and anaphase was performed based on localization, as shown in Figure 3A.

2. It would be useful if the authors described how they were able to eliminate focal plane drift (i.e., movement of a plus end into an adjacent focal plane) as a possible explanation for comet length shortening.

We thank the reviewer for raising this consideration, which we considered in our experimental design. First, we intentionally selected cell types that are known to remain flat throughout mitosis (Gorbsky et al., 1988; Rusan et al., 2001; Wadsworth et al., 2005) to minimize the risk of plus ends moving out of the focal plane. Furthermore, although movement into an adjacent focal plane cannot be entirely prevented, the average analyzed track duration is ~6 s, with average growth rates between 160–210 nm/s, yielding typical track lengths of ~1100 nm per track. Even if growth occurred entirely along the z-axis, this

would span no more than ~2 focal planes when imaged with a 1.4 NA 100× objective on a spinning disk confocal, where the axial resolution is approximately 500–800 nm. Finally, for each comet measurements, we are determining both the growth rate and the comet length and directly relating these two parameters to each other. We note that any potential growth into the z-plane would have the same effect on reducing both the measured comet decay length and the associated growth rate, as both would be equally reduced by a factor of cos θ (where θ is the angle of growth away from the xy-plane), due to the projection of the 3D trajectory onto the xy-plane. Importantly, this projection factor cancels out in the linear relationship between growth rate and comet size and therefore does not affect our ability to determine the relationship between these parameters.

3. The Conclusions section of the manuscript is strong but could be improved, e.g., by adding text recognizing that microtubule growth rates contribute to spindle length regulation (e.g., Lacroix et al. (2018); Reber et al. (2013)) as well as chromosome segregation fidelity (Ertych et al. (2014)). GTP cap size may be an important aspect of these biological phenomena. Moreover, it has been suggested that Cyclin A and Cyclin B control spindle microtubule dynamics during prometaphase versus metaphase (Kabeche and Compton (2013)). It would be interesting if the differences in GTP cap size was directly regulated by the core cell cycle machinery.

We thank the reviewer for these thoughtful suggestions. We agree that regulation of GTP cap size may play an important role in both spindle length control and chromosome segregation fidelity. We also appreciate the possibility of a direct role for core cell cycle machinery in modulating GTP-cap size and recognize this as an important avenue for future studies. We have now incorporated the suggested references and revised the accompanying text to strengthen the Conclusions section, as follows:

Lines 311-314: "In both stages, simply slowing growth would be insufficient or counterproductive as microtubule growth rate contributes to both spindle length regulation (Reber *et al.*, 2013; Lacroix *et al.*, 2018) and chromosome segregation fidelity (Ertych *et al.*, 2014)."

Lines 334-338: "Potential triggers for this modulation include signaling molecules such as Cyclins A and B, whose presence is sufficient to alter microtubule dynamics in vitro (Verde et al., 1992) and which are proposed to differentially regulate spindle microtubule dynamics across distinct stages of the cell cycle (Kabeche and Compton, 2013)."

4. It would be very helpful to insert a figure similar to Figure 1 for the analysis done in PtK1 cells.

We agree that including representative images of the PtK1 cells would be helpful. Per the reviewer's suggestion, we have added an additional Supplemental Figure S2 with representative images of PtK1 cells in each analyzed cell cycle stage.

Minor comments
1. A scale bar is missing from Figure 1F.

We thank the reviewer for identifying this omission. We have revised Figure 1 to ensure that a scale bar is present on all images.

RE: Manuscript #E26-01-0054R
TITLE: "The microtubule GTP-tubulin cap size is modulated during cell division"

Dear Dr. Zanic:

I am pleased to accept your manuscript for publication in Molecular Biology of the Cell.

Sincerely,
Alexander Mogilner
Monitoring Editor
Molecular Biology of the Cell

Dear Dr. Zanic:

Congratulations on the acceptance of your manuscript! Thank you for publishing your work in Molecular Biology of the Cell (MBoC).

Within 10 days, an unedited PDF of your manuscript will be published on MBoC in Press, an early-release journal version. The date your manuscript appears on this site is the official publication date.

Your copyedited and typeset manuscript will be scheduled for publication in the next available issue of MBoC. Once your paper is ready for review, our production team will notify you. In the summer of 2024, our production provider will introduce an online proofing solution, which will streamline the process of checking and validating author changes. This will result in improved typesetting quality, enhanced digital content, and a faster overall process.

Your paper is among those chosen by the Editorial Board for Highlights from MBoC. Hightlights from MBoC appears in the ASCB Newsletter and highlights the important articles from the most recent issue of MBoC.

All Highlights papers are also considered for the MBoC Paper of the Year. In order to be eligible for this award, however, the first author of the paper must be a student or postdoc. Please email me to indicate if this paper is eligible for Paper of the Year.

We invite you to submit images related to your accepted manuscript to be considered for the journal cover.
Cover images must contain color, TIF Format
Size image at 7.4" wide x 5.85" deep
RGB and CMYK color (please submit two separate files, one in each format)
300 dpi resolution

Please also send a 1-paragraph description of the image that a general audience could understand. Include with the caption the name and institution of the individual(s) the image should be attributed to. Please provide this description in a Word file. You can send the file via email or by your favorite file transfer service to mboc@ascb.org.

Authors of Articles and Brief Communications are also encouraged to create a short video abstract to accompany their article when it is published. These video abstracts, known as Science Sketches, are up to 2 minutes long and will be published on YouTube and then embedded in the article abstract. Science Sketch Editors on the MBoC Editorial Board will guide you as you prepare your video. Information about how to prepare and submit a video abstract is available at www.molbiolcell.org/science-sketches. Please contact mboc@ascb.org if you are interested in creating a Science Sketch.

We look forward to publishing your paper in MBoC.
Sign up for MBoC's new content alerts here https://tinyurl.com/3bcsyk3e

Sincerely,

Eric Baker
Journal Production Manager
MBoC Editorial Office
mbc@ascb.org
